# On the Combination of Acids with Animal and Vegetable Substances

**Published:** 1810-10

**Authors:** M. Thenard


### *On the Combination of Acids with Animal and Vegetable Substances. By M.* THENARD.

MY researches upon the nitric, muriatic, and acetic ethers, and upon those obtained by treating alcohol with muriate of tin and oxy-muriatic acid,† have naturally led me to examine if it was possible to form them with the other acids. I have tried the action of these acids upon alcohol; and it was in making and varying these experiments I arrived at the singular result which I have had the honour to communicate to the Institute† ; a discovery, that when the vegetable acids are pure, none of them, excepting the acetic, combine with alcohol with the loss of their acid properties: but on the contrary, when mixed with a mineral acid capable of condensing alcohol strongly, they all form with that body a combination in which their acid properties disappear, without the mineral acid taking any part in this combination.

It is evident then, that whatever may be the mode of the combination of alcohol with a vegetable or mineral acid, the alcohol produces in these compounds the effect of a true saline base.

Now the question is, whether the property of combining with acids, and also of neutralizing them, does not belong to all animal and vegetable substances. It is very possible that this is the case; for, since alcohol possesses this property, all these substances may also possess it. It was with a view to solve this question that the following experiments were made.

I passed over 300 grammes of alcohol, oxy-muriatic acid gas made from a mixture of 1750 grammes of muriate of soda, of 450 grammes of black oxide of manganese, of 800 of concentrated sulphuric acid, and of 800 grammes of water.

Almost all the acid and the principal part of the alcohol were mutually decomposed, and either generated or liberated a large quantity of water, of matter having an oily appearance, of muriatic acid, and a small quantity of carbonic acid, and a substance abounding in carbon; a result that agrees with what has already been published either by M. Berthollet in the Memoirs of the Acadsmy, or by myself in the first volume of the *Memoires d'Arcueil*. All these products have been separted with care, as I have noticed in those Memoirs; one only has been subjected to a new examination, and that is the oily matter.

* Vol. i. *des Mémoires d'Arcueil.*
† See the preceding Memoir.

When

When carefully purified by water and potash, it has the following properties, some of which have been already observed in the memoirs I shall quote. It does not redden turnsole paper; it is white; it has a cool taste similar to that of mint, and a particular but not ethereated smell; it is heavier, yet less volatile, than water; it is very soluble in alcohol, but very slightly in water. It is volatilized by distillation with nitric acid, and partly decomposed; but the products of this decomposition vary according to the strength of the acid used. If the nitric acid be weak, much muriatic acid is produced, and little oxy-muriatic: if, on the contrary, the acid be concentrated, little muriatic acid is procured, but much oxy-muriatic acid: of course this substance contains a very considerable quantity of muriatic acid. In the same manner, when it is passed through a red-hot iron tube a large quantity of acid is disengaged. Yet it is decomposed but very slowly by the strongest alkalis, even when dissolved with them in alcohol: hence the conclusion must be drawn, that the muriatic acid it contains is intimately combined with another substance. I have not yet succeeded in discovering what this substance is, because I have not been able to separate it from every thing else. Whatever that may be, it is certain that it is capable, like the alkalis, of neutralizing acids; and it may be presumed that it contains a large quantity of carbon, since in the decomposition of alcohol and oxy-muriatic acid, much water and very little carbonic acid are produced.

But of all the vegetable substances, I am acquainted with none that possess the property of uniting themselves to acids, in a more eminent degree than some of the essential oils; perhaps even all of them enjoy this property. That of turpentine absorbs nearly one-third its weight of muriatic acid gas, and becomes converted, with the emission of much heat, into an almost entire crystalline substance. Kind some years since discovered it; its nature was afterwards studied by Tromsdorff and some French philosophers, and last of all by Gehlen. All these chemists, except Gehlen, have considered it as an artificial camphor, because it had the smell, volatility, lustre, whiteness, and many other properties of natural camphor; and comparing the action of muriatic acid on oil of turpentine, with that of sulphuric acid on vegetable substances, they have conceived that the transformation of this oil into camphor is solely to be attributed to the loss of oxygen, and hydrogen being abstracted in sufficient quantity by the muriatic acid to form water, and to a slight separation of carbon at the same time; in fact, that the artificial camphor with with a little more carbon, and a certain quantity of oxygen and hydrogen in the proportions to constitute water, would return to an essential oil.

Gehlen rejected this theory for good reasons*. Having observed that in passing muriatic acid through the essential oil of turpentine, no gas was disengaged; that only a certain portion of the oil was converted into white crystals having the appearance of camphor, and that the remainder appeared as a brownish-black liquid of great acidity; that the crystals too as well as the liquid contained muriatic acid in intimate combination, &c. he has concluded, that in this operation the essential oil of turpentine is decomposed, that the greatest part of its hydrogen combines with a small quantity of its carbon, and with a certain portion of muriatic acid, to form concrete camphor, whilst the other principles of the oil combine with the remaining portion of muriatic acid to form the brownish-black liquid; therefore that this liquid contains more carbon and less hydrogen than the camphoric concretion; and that, in the formation of both, the muriatic acid acts only by the tendency it has of uniting to them, and not, as the chemists above quoted say, by forming a certain quantity of water at the expense of the principals of the essential oil.

I repeated and varied with great care the experiments on the essence of turpentine and muriatic acid, made by Kind, Tromsdorff, &c., and above all by Gehlen. I obtained the following results: 100 grammes of essence of turpentine purified by distillation, and plunged into a freezing mixture of ice and salt, absorbed 30 grammes of muriatic acid gas; no gas but muriatic escapes, which perhaps was not entirely absorbed at first, or passed through the liquid when the operation was nearly finished. The essential oil is converted into a soft and crystalline mass, from which, during its desiccation for three days, may be separated 20 grammes of a liquid that contains many crystals and nearly 100 grammes of a white substance, granular, crystaline, volatile, and having a strong smell of camphor. This substance quickly loses, by exposure to the air, the property which it at first has of reddening turnsole paper, and afterwards only very minute quantities of acid can be separated from it, even by warm alkaline solutions. Yet when it is sublimed, the vessels in which the operation is made become strongly acid; and when, instead of subliming it, it is passed through a red-hot iron tube, a

* Vide *Journal de Gehlen*, tome vi. p. 458.

greater quantity of acid is still abstracted from it. Lastly, when decomposed both by concentrated and weak nitric acid, a large quantity of oxy-muriatic acid is produced in the first instance, and in the second much muriatic acid. Relative to that portion of liquid derived from the crystalline mass, the weight of which was 20 grammes, it was white, and diffused acid vapours, but ceased after an exposure of some days to the atmosphere, and was no longer acid; it crystallized in mass some degrees below zero, and preserved an intimate union with much muriatic acid.

Thus all my observations, except one only, agree with those of Gehlen. That in which we do not coincide is relative to the liquid product of the operation. Gehlen obtained it of a brownish-black, and I always obtained it white. This difference, I think, depends on Gehlen having used the essence known in commerce, which contains, unless distilled with great care, a resin, and has then the property of blackening and forming a large uncrystallizable residue. Neither do I draw from my observations the same theoretical inferences as this chemist does from his. I think that the essential oil of turpentine is not decomposed by muriatic acid, and consequently that these two bodies combined together in certain proportions form concrete camphor; probably the small portion of liquid product obtained by him is of the same formation; besides, it is not improbable that the difference existing between the camphoric concretion and the liquid product, depends on a small quantity of an ethereal oil mixed with the essence, and capable of forming with muriatic acid a liquid product. Lastly, what induces me to believe that the muriatic acid does not decompose the essence, but, on the contrary, combines with it, is, that this acid evidently combines with all the principles of alcohol without affording many different products, and that alcohol is a hydrogenous body as well as the essential oil of turpentine. Yet whatever it may be, nothing is more certain than that this combination has the greatest resemblance to native camphor.

Perhaps the camphor which is extracted from the essential oils of plants, particularly from that of lavender, is a combination of essence and acid alone; perhaps too the camphor of commerce, or the camphor of the laurel, which does not appear to contain any mineral acid, (for none can be obtained from it by destructive distillation,) is formed of an essential oil and vegetable acid alone. These views, as they are founded on facts, at least deserve some attention, and, if confirmed by experience, may probably be of great advantage.

I have

I have likewise formed the combinations of the essence of lemon and of lavender with muriatic acid. 32 grammes of the essence of lavender require 22 of acid; the resulting compound is blackish, acid and liquid. 26 grammes of the essence of lemon absorb 22 grammes of acid; the essence is of a brownish hue and solid. Neither of these essences had been purified; but both were used in the state they are generally found in commerce.

The essential are not the only oils capable of combining with acids. It has long been remarked that the fat oils form with different acids peculiar kinds of soap. The sulphuric acid when concentrated has this effect upon all of them; it produces with the oil of olives in particular a soapy matter, of a thick and greenish appearance, which gradually acquires consistence; when washed with water it becomes white; it is acid, but less so than if the acid was uncombined, and it may be brought into the neutral state by a proper quantity of potash.

Tannin besides, which is a vegetable matter, contracts, as is well known, a strong union with acids; for the concentrated sulphuric acid suddenly precipitates it from its solution in water, and I am convinced that however often the precipitate may be washed, it is always acid. Nature itself has offered us a compound of this species. In fact, I have treated 10 grammes of gallnuts with 2 litres of boiling water, and although the residue had been washed with two litres more of very warm water, it still strongly reddened tincture of turnsole: in the gallnuts therefore the gallic acid is retained by a true affinity. But as the quantity of gallic acid abstracted by a spontaneous decomposition far exceeds the quantity of free acid existing in its natural state, we must conclude that the greatest part of the acid is certainly neutralized by the tannin. It is observed too, that if a decoction of gallnuts be allowed to follow its own course, as the tannin is destroyed, the liquor becomes more and more acid. One may, indeed, attribute this phænomenon to the transformation of the tannin itself into gallic acid; but numberless observations authorize us to believe that this is not the case.

1. That vegetable substances during their decomposition form no other acid but the acetic.

2. That gallnuts evidently contain abundance of gallic acid, and that, this acid having a strong affinity for tannin, the two bodies should necessarily neutralize each other in part.

3. Because there are many remarkable instances of the power of some vegetable substances to neutralize acids.

4. Lastly,

4. Lastly, that tannin precipitated from a decoction of gallnuts by an alkali or carbonate of ammonia, contains, according to my experiments, a good deal of gallic acid as well as a small portion of saline base. The proof I have of this is, that it produces with almost all the metallic solutions the same effects as gallic acid, or gallate of potash. Thus it gives red precipitates with solutions of the peroxides of mercury, blackish gray with solutions of peroxides of iron, a blue one to those less oxidized, the colour of wine lees *(lie de vin)* to those least oxidized. It follows then that we are not yet acquainted with the pure tannin of gallnuts, and of most other substances, and consequently that we know not how to act with them on metallic solutions, &c. Inquiries which have this object in view cannot but be interesting; and I propose to pursue them, in as much as they will on some future day allow me to bring forward the question I am now treating of.

If, after having examined the vegetable substances which readily combine with acids, a search is made among animal substances endowed with the same properties, five will evidently occur: curdy matter, albumen, *picromel*, gelatin, and urea. This property is generally found in the curdy matter, as it is known that acids coagulate milk, that the coagulum contains an acid, and that this acid is even sensible to turnsole paper. Of course the affinity between those two bodies is well marked; yet it is not sufficiently strong to destroy all doubts to the contrary; for I am satisfied that the excess, and perhaps also the whole, of the acid may be abstracted by means of a large quantity of water.

If water alone be sufficient to take the acid from the curdy matter, it fails to produce the same effect when the acid is united to albumen; for the washings may be continued to an unlimited extent, and there will be always found acid undissolved.

We must conclude therefore, that this animal substance exerts on acids a more powerful attraction than the curdy matter; but this action varies in itself according to the concentrated state of the acid. As the acid is saturated with water, the results are combinations more or less insoluble, and which again dissolve, the acid being saturated with ammonia, or with either of the other alkalis, and in which albumen is nowise altered. If on the contrary the acid is very strong and concentrated, the precipitates formed will always be acid, but will contain albumen in the state in which it exists when concreted by fire: this has appeared in treating the precipitates with a weak solution of ammonia. In the first instance, the solution solution will be gradually formed and complete; in the second, the acid alone will be taken up, and the albumen will remain undissolved in the form of a thick *magma*.

The combination of picromel with the acids took place with the same facility as with the preceding. Almost as soon as these substances come in contact, an acid precipitate is formed, which redissolves when neutralized. It is principally with the sulphuric, nitric, and muriatic acids that picromel forms slightly soluble compounds.

The concentrated nitric acid is the only one, according to Messrs. Fourcroy and Vauquelin, that precipitates urea from its solution in water; yet it is probable that the other acids are capable of combining with it; and if they do not precipitate it, it is because the compounds are soluble in water. I have observed that these combinations never form when the temperature is below 40 or 50, particularly with the sulphuric and muriatic acids; and that otherwise there is a disengagement of carbonic acid, and a production of a large quantity of ammonia.

Lastly, gelatin itself may unite with some acids, and principally with the oxy-muriatic acid. To effect this combination it is necessary to pass the gaseous acid through a solution of gelatin. In this operation the solution gradually becomes turpid and precipitates flakes, which unite together in the form of pearly filaments very elastic and flexible. These flakes have been considered by M. Bouillon-Lagrange, in a memoir upon the gizzard of birds, as the oxygenated gelatin *: but it is a mistake; they are really formed of gelatin slightly altered, and of the muriatic and oxy-muriatic acids. Their striking characteristics are, that they are insipid, insoluble both in water and alcohol, not liable to putrefy, slighty acid; though a sufficiently large portion of acid enters their composition to emit spontaneously for several days the oxy-muriatic acid gas, much more may be disengaged by means of heat: lastly, they contain so much acid as to be soluble in alkalis, and to form with them muriates.

Thus we have examined five vegetable matters, and six animal matters, capable of an intimate union with acids. Three of the former, viz. alcohol, essential oil of turpentine, and a substance abounding in carbon, and produced from alcohol decomposed by oxy-muriatic acid, neutralize acids equally as well as the strongest alkalis. The last seven form

* *Annales de Chimie*, tom. lvi. p. 24.

with with these acids combinations, which are themselves acid, like the metallic and most of the earthy salts.

Without doubt we shall in time be able to combine all other vegetable and animal substances with acids, and the experiments related allow even at present the inference to be drawn: for, if the combination cannot be made directly, no proof appears in this against what I advance. Does not alcohol, which in its ordinary state has not the property of neutralizing the vegetable acids, acquire it by the presence of a mineral acid? When these substances are placed in different circumstances, probably, the one best adapted to unite them with the acids will be found. These researches are laborious indeed; but they are useful and important, as tending to introduce us to the knowledge of a long compound series of a particular order; a knowledge which in itself must throw great light upon the analysis of vegetable and animal substances. In fact, is it not possible that we may find in organic bodies compounds of this kind? Are not gallnuts an example? Is it not possible that acetic acid, which we procure by the distillation of animal and vegetable substances, may be contained ready formed in some? Amber in particular, from which we get by distillation succinic acid, is it formed of an oily matter combined with succinic acid? The fatty substances which afford us sebacic acid, may they not also be in similar circumstances? Lastly, the bitter principle formed by treating animal substances with nitric acid, which acts as a very oxygenated body, although not acid? and the yellow acid, which, according to MM. Fourcroy and Vauquelin, is formed in the same operation, may they not be intimate combinations of an acid and of another matter? But it is above all things necessary, in the explanation of phænomena which pass before us, in the decomposition of vegetable and animal substances by acids, that a strict account should be kept of their tendency to unite with these bodies.

CRITICAL

